# A novel deep unsupervised approach for super-resolution of remote sensing hyperspectral image using gompertz-function convergence war accelerometric-optimization generative adversarial network (GF-CWAO-GAN)

**DOI:** 10.1038/s41598-024-81163-x

**Published:** 2024-12-02

**Authors:** K. Deepthi, Aditya K. Shastry, E. Naresh

**Affiliations:** 1 Information Science & Engineering, Nitte Meenakshi Institute of Technology, Visvesvaraya Technological University, Bengaluru, India; 2 Department of Information Technology, Manipal Institute of Technology Bengaluru, Manipal Academy of Higher Education, Manipal, India

**Keywords:** Super-resolution (SR), Low resolution (LR) image, High resolution (HR) image, Remote sensing image, Generative Adversarial Network (GAN), Shannon Gaussian Filter, Gompertz Function, Accelerometric optimization, PSNR, SSIM, Inception score, Fréchet Inception Distance, Environmental social sciences, Solid Earth sciences, Engineering

## Abstract

Hyperspectral remote sensing images obtained from cameras are characterized by high-dimensions and low quality, which makes them unfavorable for various analytics purposes. This is due to the presence of visible and invisible frequencies of the reflected light making it poorly reveal the spectral signatures of the image. Visual communication advancement has paved the need for Image Super-Resolution (SR) which recovers high-resolution images from low-resolution images. Several works were carried out earlier on image SR using variants of supervised and unsupervised models that still lack accuracy. In this paper, we propose an unsupervised learning model titled Gompertz Function–based Convergence War Accelerometric Optimization–GAN framework for generating of High-Resolution (HR) images. The framework comprises a pre-processing stage, where the incoming Low-Resolution (LR) image is preprocessed for noise removal by applying Shannon-Gaussian Filter (S-GF). Following is the Gradient Domain Approach based Tone-Mapping (TM). Skew correction is done to remove distortion and maintain original resolution that may change during TM stage. The next stage comprises the boundary and edge enhancement of the resulting preprocessed image generated by the method of Inverse Gradient Mapping (IGM) followed by patch extraction to extract minute low-frequency information from the resulting boundary and edge-enhanced image. The contrast of the enhanced patches is improved by removing blurriness effect. The preprocessed image patches are then fed into the Gompertz Function-based Convergence War Accelerometric Optimization – GAN for feature mapping on the trained SR Image features that are clustered using Krzanowski and Li- Kantorovich Metric-K-Means clustering Algorithm (KL-KM-KMA) for effective generation of SR image. The developed model is validated for both qualitative and quantitative measurements. Comparisons are made with several other state-of -the-art methods for accuracy of 98.05%, precision of 97.98%, inception score of 8.71, Fréchet Inception Distance of 36.4 with reduced clustering and training time proving the efficiency of the proposed model.

## Introduction

Remote sensing images attract the attention of various fields like environment monitoring, remote sensing and computer vision. Hence, in the process of exploring the Earth, obtaining HR image of Earth is of great significance for scientific research. However, due to the variations in temperature, atmospheric pressure and other environmental conditions on Earth, the images obtained from hyperspectral cameras are unstable and most of the times blurry, obscure thereby reducing the usability of data. Image SR aims to facilitate better visualization of remote sensing images thereby addressing the challenges associated with a wide range of real-world applications.

Deep learning-based image SR of remote sensing images has gained importance in recent years. There are several conventional SR methods that are based on Interpolation often require extensive computational resources and enormous training time, hindering their practical implementation under resource-constrained environment. From the literature, it is prevalent that most of the earlier work proposed on image SR was supervised method based that demands huge paired LR-HR images for training and posed a huge challenge in accumulating huge data set. Several unsupervised approaches were also proposed that provide relaxation with respect to data set size and unavailability of paired LR-HR images.

Recent advances in Convolutional Neural Network (CNN) addressing SR have progressed in image SR, on the other hand due to ill-posed nature of SR images generated, the existing Convolutional Neural Network oriented techniques often loose texture details thereby causing excessive smoothing of image^1,2^. To generate more visually realistic SR images, Unsupervised Generative Adversarial Networks (GANs), their variants and hybrid GANs can be used and have affirmed to accomplish superior super-resolved satellite images^3^. Although GAN-based approaches achieve SR of satellite photos, there are several challenges that are still prevalent. Firstly, they often struggle with generalization across diverse datasets leading to performance inconsistencies. Secondly, training instability leads to generators producing limited outputs affecting the quality of SR images. Existing works also failed to preserve the low frequency information during image SR generation step. Third, there is a gap in exploring hybrid GAN architecture specifically designed to improve SR performance. Lastly, Unsupervised GANs are computationally intensive, leading to scalability issues in real-time applications suggesting a need for more efficient hybrid architectures, advanced algorithms and techniques.

To solve the above-mentioned problems, an efficient image super resolution model using GF-CWAO-GAN based image SR technique is proposed to perform effective matching of feature points resulting in better SR image generation. Patch extraction and Inverse Gradient Matching are introduced for better preservation of low frequency information and image characteristics respectively. Specifically, the model also stands better in effective preprocessing of input image by the introduction of Tone Mapping and Skew Correction task for preserving both high and low frequency information. Extensive experimental results justify that the proposed GF-CWAO-GAN obtains acceptable performance than existing traditional, CNN and GAN-based methods qualitatively and quantitatively.

The contribution of the current article can be made as follows:

- A framework named GF-CWAO-GAN is proposed that significantly improves the grade of SR reformation of Hyperspectral Remote-sensing image.
- An improved strategy for Pre-processing Step for extracting determinant features/pixels inclusive of Boundary and Edge Enhancement, Patch Extraction and Patch Enhancement is proposed to effectively enhance the features extraction process for SR reconstruction. Krzanowski and Li-Kantorovich Metric-K-Means clustering Algorithm is proposed during the training phase followed by further effective generation of SR image with GF function in the Generator Model and initial matching and propagation matching in the Discriminator Model.
- The Fréchet Inception Distance was introduced to measure the loss between real and generated images to improve the spatial-spectral characteristics in reconstructed SR hyperspectral remote-sensing image.

The rest of the work is organized as follows. In Sect. 2, the earlier work and issues related to proposed work is presented. Section 3 provides the framework/network architecture of the proposed model. Section 4 provides algorithms, experiments and theoretical analysis of model parameters and loss. In Sect. 5, experimental results along with their quantitative and qualitative analysis are presented. Finally, the article is concluded with a conclusion.

### Related work

Image Super-Resolution (Image SR) refers to the process of obtaining HR image from LR image retaining high quality, sharp edges and wanting for more pixels with very few artifacts. according to previous literature, SR methods are classified as Single-Image SR(SISR) and Multi-Image SR(MISR)^4^. SR can do either using supervised or unsupervised methods using CNNs^5,6^and GANs. However, GANs provide powerful framework and outperform CNNs in unsupervised SR tasks by leveraging adversarial learning and focusing on perceptual image quality in generating realistic images. The SR methods are applied to the various image types like medical images^7^, remote sensing images^6,8–10^, hyperspectral images^11^, realistic images^4^and other types of images. The literature overview on SR is included in^12,13^. This section conducts brief literature review on GAN-based SISR models for satellite image SR and their related issues recapitulated as below.

Ever since Ian Good Fellow et al., proposed GAN in^14^, they are rigorously studied by several researchers as detailed below. Several attempts on employing GANs for unsupervised learning in recent years. Adoption of GAN-based Deep Learning SR models for remote sensing image SR has gained increasing popularity in recent years, each one offering some improvement in terms of accuracy and resolution produced. Recent advancements in utilizing GANs to upgrade the SR of Mars image, include novel two step framework incorporating new degradation framework. It is designed to estimate blur kernels and GAN trained to generate noise distribution over Mars2K dataset^15^.

According to^8^, Multiattention GAN(MA-GAN) comprises of three blocks to automatically learn and perform scale adjustments for better image representation. The channel attention (CA) and pixel attention (PA) upscale LR images to HR images taking adversarial loss and pixel loss as guiding parameters. Cascade GAN(CGAN) addressed the issue of uncontrollable performance of discriminator and unstable training by introduction of content fidelity to avoid vanishing gradient and scene constraint to achieve high quality SR image^3^.

A GAN-based approach has demonstrated superior performance for hyperspectral image SR is proposed with residual learning incorporated to achieve spectral fidelity utilizing gradient features as auxiliary information to carry out counter training with discriminator^11^. Shaolei Zhang et al., 2021 had put forward an unsupervised GAN approach to hyperspectral image SR by group convolution and attention mechanism via degradation learning on the public dataset CAVE and have justified to outperform the SR ability over other models^16^.

According to^9^, Yingfei Xiong et al., proposed improved SRGAN (ISRGAN) with stable training and enhanced generalization abilities across locations and two sensors Landsat 8 OLI and Chinese GF1 with improved PSNR (35.816) and SSIM (0.988) parameters. Also, Radford et al., proposed Deep Convolutional GAN where GANs performed feature extraction to capture semantic content from the images enabled with vector arithmetic^17^.

The unsupervised objective of image SR is realized focusing on working principle of GAN. In principle, the GAN architecture design comprises of generator plus discriminator are trained together/simultaneous with competing aims. While the generator network attempts to generate images that are indistinguishable from natural images, the discriminator network is trained to discriminate between original image and synthesized image.

Wang et al., in^18^ proposed ESRGAN, a groundbreaking approach in SISR prioritizing perceptual quality over PSNR (traditional metric). ESRGAN is an enhanced version of SRGAN with Residual-in-Residual Dense Blocks (RRDBs) supporting improved feature extraction, removes Batch Normalization layers to avoid artifacts. More realistic image generation was observed using relativistic discriminator inception.

Several clustering algorithms were seen in earlier literature. James c et al., proposed Fuzzy C-means Clustering (FCM) that generates fuzzy partitions and prototypes useful for suggesting substructures in unexplored data using least-square objective function^19^. In^20^, researchers achieved contrast improvements using Mean Shift (MS) clustering resulting in greater accuracy. Tian Zhang et al., proposed data clustering technique titled BIRCH (Balanced Iterative Reducing and Clustering using Hierarchies) to generate best quality clusters via incremental and dynamic cluster formation of multidimensional data points^21^. Several researchers have used K-Means Clustering algorithm and their variants for identifying clusters.

From the thorough study of the earlier works, it is prevalent that they faced the issues and challenges in terms of loss of naturalness in generated images, convergence, extensive need for computational resources while training, lack of generalization and so on. In summary, all the unsupervised GANs discussed above are computationally intensive and resource constrained. The current work proposed focusses on resource efficient GF-CWAO-GAN architecture and leverages Google Colab’s TPUs for accessibility.

### The proposed GF-CWAO-GAN framework/proposed network architecture

The aim of remote sensing hyperspectral image super-resolution is to obtain high resolution image from low resolution image to improve the visual communication in various fields like satellite and astronomical imaging, military surveillance, defense and remote sensing. Hence, this paper presents an efficient novel deep learning-based GF-CWAO-Generative Adversarial Network (GF-CWAO-GAN) based framework to generate super-resolution images.

### Basic network architecture

**Fig. 1:**
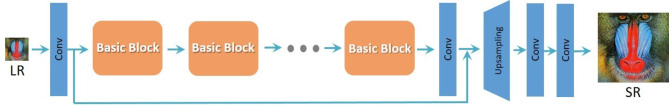
Basic ESRGAN Architecture.

The original ESRGAN Architecture is as shown in the Fig. 1 below which forms the basis for the proposed hybrid network architecture model.

The overall configuration is retained consisting of Generator and Discriminator networks as proposed in^18^.

### Proposed architecture of GF-CWAO-GAN

**Fig. 2:**
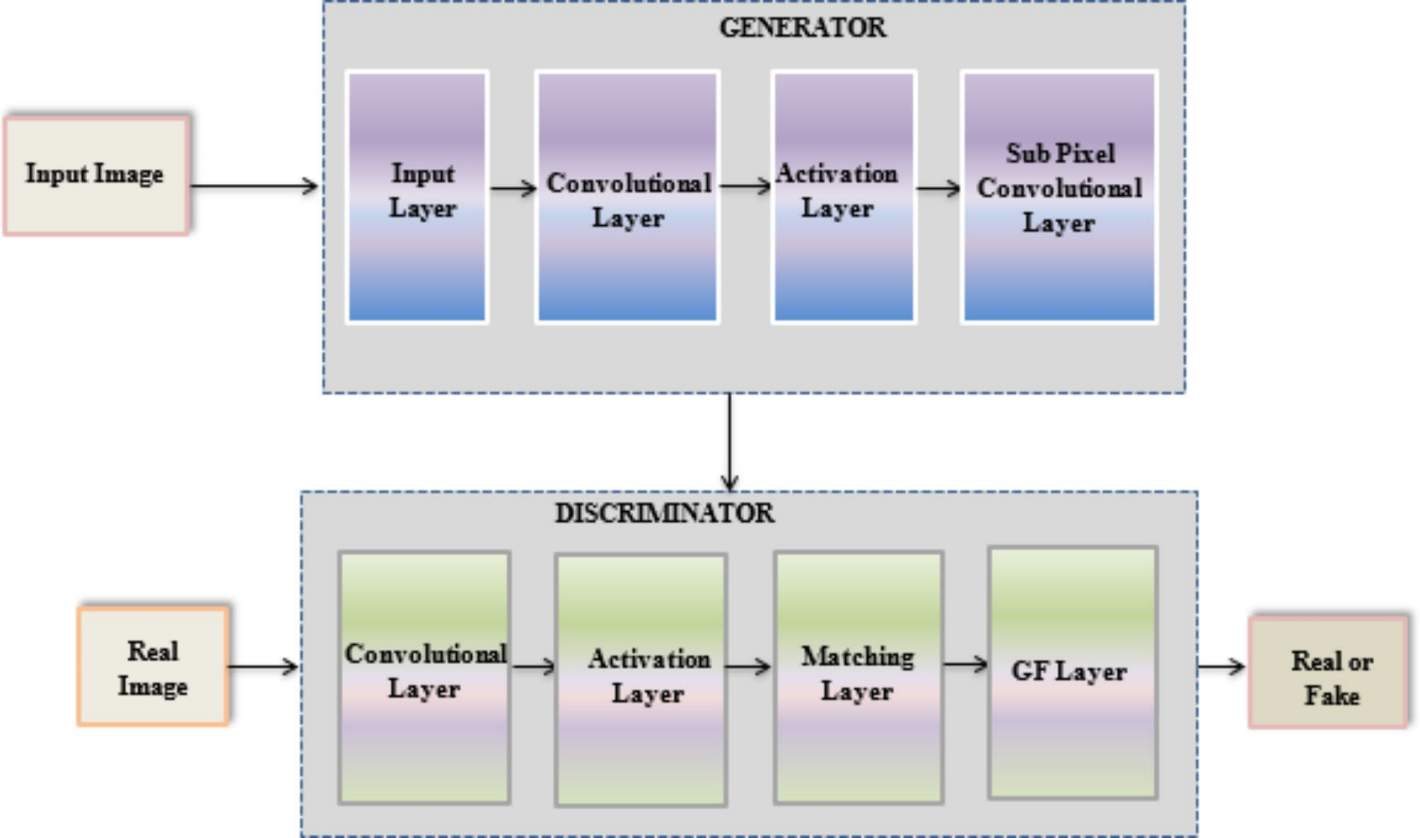
Proposed GF-CWAO-GAN Model.

Super-Resolution images are always of interest as they conserve additional and crucial details playing a key role in various fields like medical imaging, astrophotography, surveillance and others. Various deep learning-based frameworks are proposed by several researchers and Generative Adversarial Networks (GAN) has played a prominent role in Image Super-Resolution. Inspirited by the remarkable performance of GANs under unsupervised learning, herewith we propose Gompertz Function – Convergence War Accelerometric Optimization GAN (GF-CWAO-GAN) with densely-connected CNN generator network and modified Discriminator framework. Earlier GAN as proposed by Z Wang et al., 2020 used sigmoid activation function at discriminator resulted in gradient diffusion problem and incorporation of Wasserstein distance metric for measuring loss between generated and original image increased the distribution loss. To overcome this drawback, in the proposed model Gompertz Function and Fréchet Inception distance are considered to reduce the gradient diffusion problem and distribution loss respectively. Hence the name Gompertz Function GAN (GF GAN). The framework for the proposed GAN is depicted in Fig. 2 below:

### Proposed architecture modifications for discriminator in GF-CWAO-GAN

**Fig. 3:**
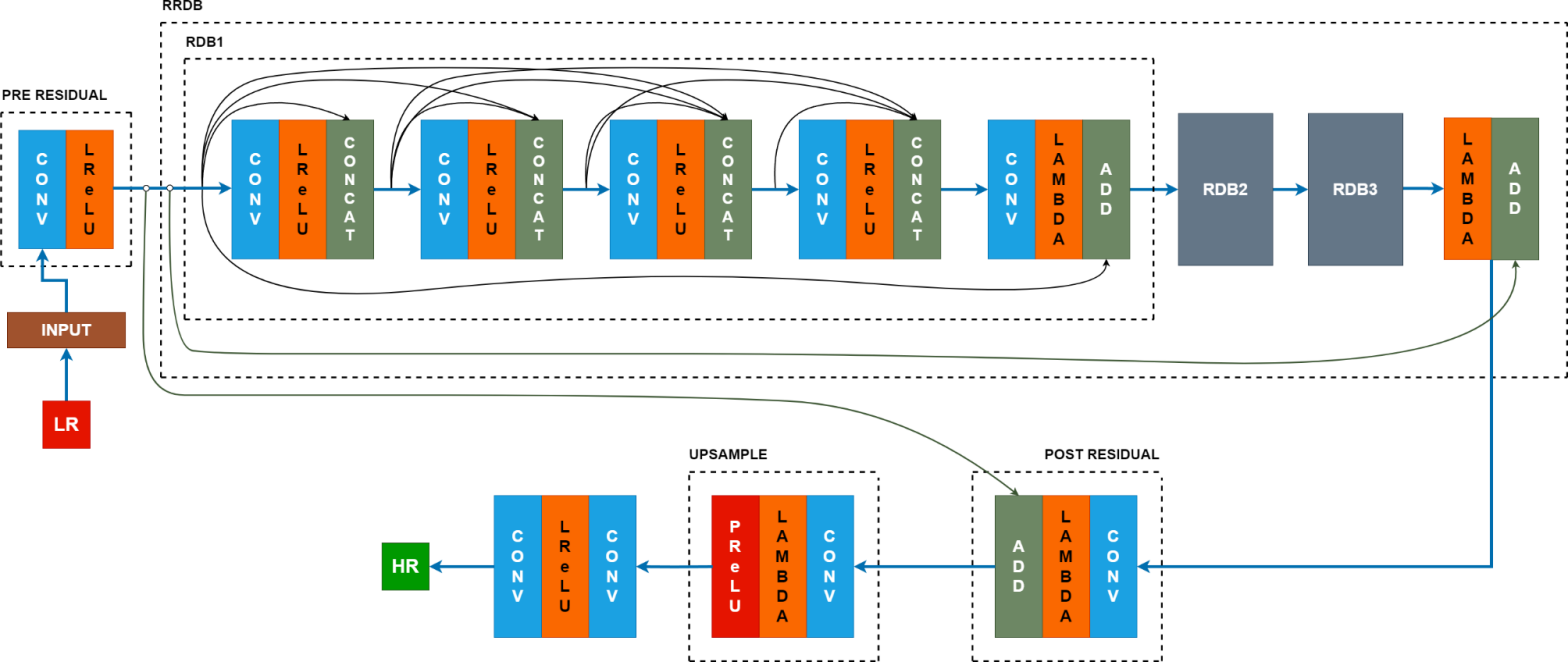
Proposed Architecture modifications for Discriminator in GF-CWAO GAN.

The proposed model implements several modifications in Discriminator to bring novelty as shown in the Fig. 3 below:

The model description of Discriminator is detailed below:

Introduction of PatchGAN as a discriminator network consisting of comprising of 8 blocks of Conv2D layer, followed by LeakyReLU layer with each block having Batch Norm layer except for the initial layer. This is done to concentrate on local image characteristics rather than overall image details, by reading images in patches.

Pre-residual block: This block is introduced before the main RDB network in the generator network comprising of convolutional layer followed by activation function, LeakyReLu for preliminary feature extraction, processing the input LR image and generating initial feature maps.

Enhanced Residual Dense Blocks: Within each RDB, Leaky ReLu activation layer is added after each convolutional layer as shown in the figure above. This activation function permits for small positive gradient even for non-positive inputs, avoiding the “dying ReLu” problem^22^. Following this is the concatenation layer for feature fusion at each step^23,24^.

Lambda and Addition layer: A mechanism is introduced to allow the network to learn the difference between the input and the processed layer. The Lambda layer followed by addition layers does this mechanism by performing identity operations and creating skip connections respectively.

Post-residual Block: This block consisting of Conv2D layer followed by LeakyReLu layer is introduced following the final RDB block in the generator network that mirrors the pre-residual block to help improve final feature representation before feeding it to up-sampling block.

Sub-pixel Convolution for Up-Sampling: Instead of nearest neighbour interpolation for up-sampling, the proposed model implements sub-pixel convolution that allows generating HR image outputs while reducing artifacts, leading to sharper and more visually appealing images.

Reduced RDBs and RDB Blocks: This is one of the major modifications proposed in the current work in which the number of RDBs and RRDB blocks are reduced compared to original ESRGAN model. The number of Residual Dense Blocks (RDBs) and Residual in Residual Dense Blocks (RRDBs) are reduced from 16 RDBs and 23 RRDBs to 1 RRDB and 4 RDBs respectively. Despite of its smaller size, it showed superior performance over the existing GANs.

### Super-resolution image generation flow diagram

**Fig. 4:**
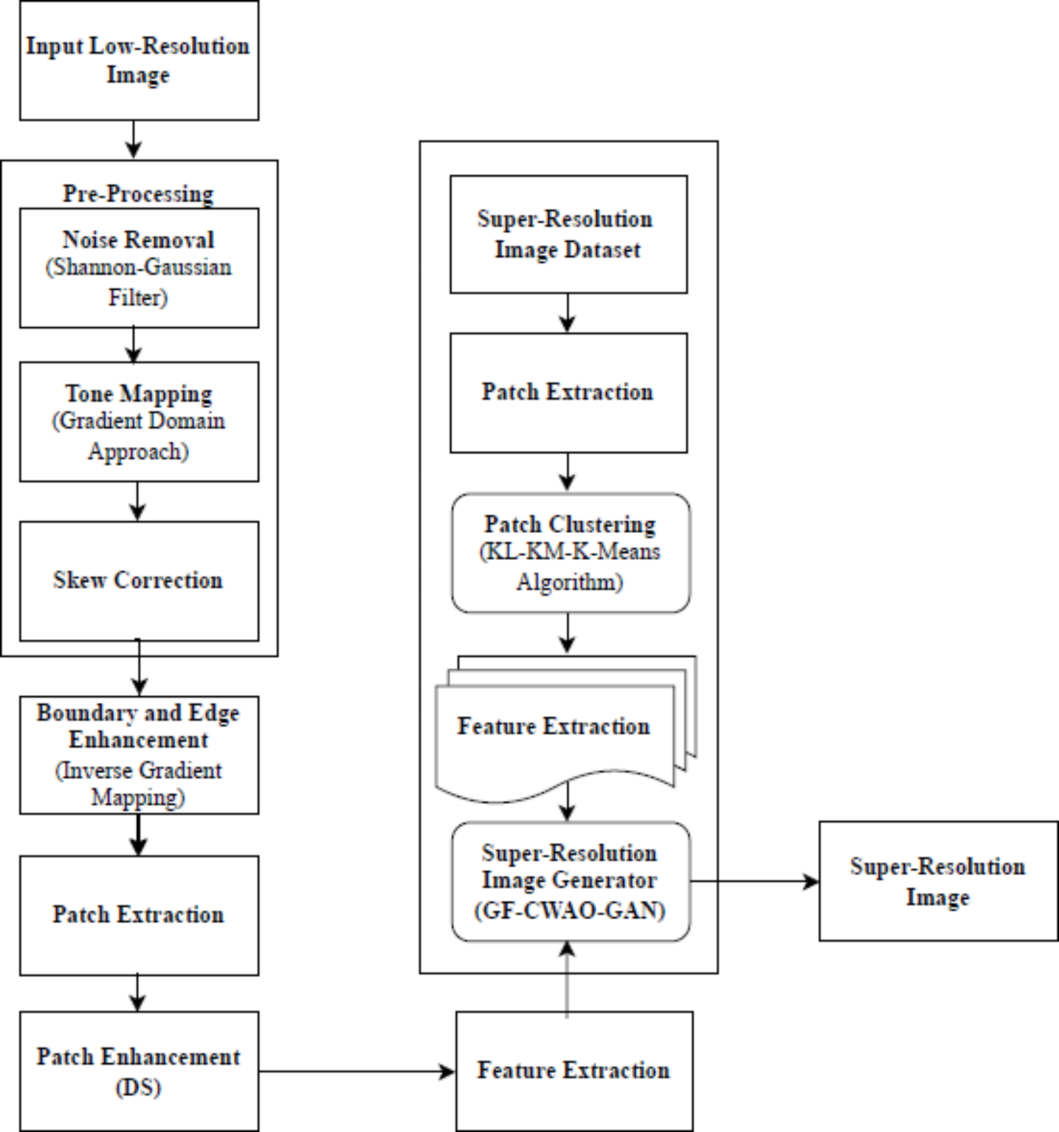
Flow Diagram of Super-Resolution Image generation process.

Figure 4 below shows the sequence of phases involved in SR image generation. In the first phase, the data is pre-processed and prepared by removing noise while preserving the original pixel intensity and reshaped to serve the purpose of research work. Following it by Boundary and Edge Enhancement, Patch Extraction, Patch Enhancement to make the image apt for Feature Extraction. The dimensions of the images are resized to [128, 128] for the testing purpose. In the second phase, the super-resolution image generation takes place with the GF-CWAO-GAN that suits the image requirements for various application fields like land cover land use monitoring, structural mapping, environmental geology, weather forecasting, mineral exploration, land cover classification etc., In the final phase, qualitative and quantitative measurements are performed to prove the effectiveness, feasibility and standard of the proposed framework.

#### Pre-processing phase

To get started with, the input LR image is pre-processed for the removal of unwanted data infused in it. The purpose of preprocessing is to generally preserve the determining/important details in each image pixel while successfully eliminating the noise component present in the image. The mathematical model of input low-resolution image is given in Eq. (1).

1 $$\:{P}_{i}\left(x,y\right)={\sum\:}_{i=1c}^{n}{P}_{i}\left(x,y\right)+{{\aleph\:}}_{0}$$

Where $\:{P}_{i}\left(x,y\right)$ is the input low-resolution image, $\:\left(x,y\right)\:$being the pixel value in the image and $\:{\aleph\:}_{0}$ is the noise component present in the image. Several pre-processing stages are involved are outlined as given below:

#### Shannon – Gaussian Filter (S-GF) based noise removal

Initially, the low-resolution image $\:{P}_{i}\left(x,y\right)$ goes through the noise removal stage to eliminate the noise found in the LR image. Noise present in the image impairs the image’s quality by manipulating individual pixel value. As a result, S-GF is applied for noise removal to enhance the quality of the image. Gaussian filter is a linear filter used in image processing either to bring blurriness in image or to remove noise from the image. In our proposed work, such a filter is used as it has effective noise filtering capability and is faster due to multiplying and addition over the median filters which are based on sorting. The degree of randomness in the image without information loss is measured by the introduction of Shannon Entropy. The integration of Shannon Entropy into Gaussian Filter is the emergence of the term Shannon – Gaussian Filter(S-GF) and given in the mathematical Eq. (2) below:

2 $$\:{P}_{S-GF}\left(x,y\right)=\frac{\pi\:}{\sqrt{2\mu\:}}{log}\left({\left({P}_{i}\left(x,y\right)\right)}^{2}/2{\mu\:}^{2}\right)$$

Where $\:{P}_{S-GF}\left(x,y\right)$ denotes Shannon – Gaussian function,

$\:\mu\:$ for Gaussian Filter variance

The outcome of this step is the noise removed image denoted as P_NR_($\:x,y).$

#### Tone mapping: gradient domain based approach

Noise removed image is at high dynamic range and to maintain and preserve original pixel intensity, gradient domain – based tone mapping is performed to output low dynamic range satellite image. The gradient domain tone mapping method operates on the assumption that the human visual system is more receptive to local intensity variations rather than absolute brightness values. These variations are accurately represented using gradients.

Firstly, the choice of image gradient (∂_ldr_) is done. The gradient value is computed by taking the finite difference between the pixels in the image and is represented as shown below:

3 $${\partial\:}_{ldr}=\text{Difference}(P_{NR}(x,y))$$

Where Difference() is the finite difference function.

Then obtained gradient outcome value is used to generate low dynamic range image (LDR), P_LDR_($\:x,y)$ by resolving the following Poisson’s model below:

4 $$\:{P}_{LDR}\left(x,y\right)={\partial\:}_{ldr}\left({P}_{NR}\left(x,y\right)\right)$$

#### Skew correction

Skew Correction is the next part after the tone mapped image is obtained. Skewing in image is something that is not exact. It indicates the amount of distortion in the image. Hence the skew correction is carried out on tone mapped image $\:{P}_{LDR}\left(x,y\right)$ to retain distortion while removing distortion in the image. The skew correction is mathematically modelled as Eq. (5) given below:

5 $$\:{P}_{sc}\left(x,y\right)=\frac{3\left(\sigma\:-\gamma\:\right)}{y}\left({P}_{LDR}\left(x,y\right)\right)$$

Where, σ represents the mean value of tone mapped image, γ and $\:y$ projects to the median and standard deviation of tone mapped image.

#### Edge and boundary detection – inverse gradient mapping (IGM) based

An edge in an image is the connection of a set of pixels that forms a boundary between any two disjoint boundaries. After pre-processing stage, the preprocessed image is enhanced to help better generate super-resolution image. Gradient Mapping (GM) is more suitable for boundary and edge enhancement with a better enhancement rate due to implementation of convolution operation. IGM is developed using four components like Superficial Feature Extraction (SFE), Local Feature Extraction (LFE), Global Feature Extraction (GFE) and Upsampling module. The following subsections highlight the above components:

#### Superficial feature extraction (SFE) component

This component aims at the extraction of superficial features (SF_f_) from the pre-processed image, $\:{P}_{sc}\left(x,y\right)$. It is given by the equation:

6 $$\:{SF}_{f}=R\left(invconv\left(invconv\left({P}_{sc}\left(x,y\right)\right)\right)\right)$$

Where R refers to parametric function, $\:invconv\left(\right)$ is the inverse convolution operation performed for edge and boundary enhancement.

#### Local feature extraction (LFE) module

The superficial features extracted from the SFE component is input to LFE module where local/neighboring features (Nf) are extracted through multiple Residual Dense Blocks (RDB) plugged in it and is represented as,

7 $$\:{N}_{f}=rdb\left({SF}_{f}\right)$$

Where rdb() denotes Residual Dense Block.

The superficial and shallow features extracted are then super-imposed to produce resultant merged parameters/features as indicated by

8 $$\:{simp}_{f}=R\left(conv\left(concat\left({SF}_{f},{N}_{f}\right)\right)\right)$$

Where $\:{simp}_{f}$ is a super-imposed feature, $\:concat\left(\right)$ is concatenation operation.

#### Global feature extraction (GFE) module

Global features denote the features extracted from the overall image whereas local features are the features extracted from neighbouring pixels of the given sub image. The resultant coalesced features are then merged with the features extracted from pre-processed image, $\:{P}_{sc}\left(x,y\right)\:$to obtain global feature, G_f_, represented as,

9 $$\:{G}_{f}={f}^{-1}+{simp}_{f}$$

Where $\:{f}^{-1}$ is input image feature.

Hence, based on the extracted features, the size and scale of the unit capable of distinguishing objects in the image is maintained by the process of up sampling. Based on the resultant slope/gradient characteristics, the boundary and edge of image is characterized, and Boundary and Edge enhanced image is denoted as $\:{P}_{BE}\left(x,y\right)$.

#### Patch extraction

Followed by the boundary and edge enhancement step, the next step is the patch extraction, a vital pre-processing step in image processing is done from the boundary and edge enhanced image. Patch in image is a group of pixels of interest in an image. Patch extraction portrays an image as a set of several sub-images (Patches) of interest. Patch extraction is done to recover the low-frequency information as defined in mathematical equation below:

10 $$\:PE\left({P}_{BE}\left(x,y\right)\right)=PE{\left({P}_{BE}^{q}\left(x,y\right)|q\right)}^{\raisebox{1ex}{$1$}\!\left/\:\!\raisebox{-1ex}{$s$}\right.}$$

Where, $\:{P}_{BE}^{q}$ is a patch of size, s, extracted from edge and boundary enhanced image.

Following it is similarity measurement task is carried out by the method of probability multiplication made on local pixels, written as,

11 $$\:{\wp\:}=\alpha\:\left(PE\left({P}_{BE}\left(x,y\right)\right)\right)$$

Where, $\:\alpha\:$ is the probability of multiplication function. It gives the probability of occurrence of pixel value in the resulting patch using the probability of occurrence of individual pixels.

The resulting patch extracted image is denoted as $\:{P}_{PE}\left(x,y\right)$.

#### Patch enhancement

During the patch extraction step, there is a possibility of contrast reduction of image. Hence to overcome this problem, further, the extracted patches are enhanced for the betterment of the image during the image generation process. In the proposed method, the Distributed Signal (DS) processing is used for efficient patch enhancement without the knowledge of the entire image.

Generally, the colors in the extracted patches are distributed and in Distributed Signal, Karhunen-Loeve Transform (KLT) is applied to these distributed colors that diagonalizes the correlation between the several distributed colors in the extracted patches after the patch extraction process. The KLT function is modeled as below:

12 $$\:{P}_{KLT}\left(x,y\right)={\sum\:}_{n=1}^{{\infty\:}}{V}_{n}{PE}_{P}\left(x,y\right)$$

Where, $\:{V}_{n}$ is termed as random variable.

Further, post correlation determination, the contrast of the distributed colors are expanded to optimize the variance in the colors. Hence, the resultant patch enhanced image is obtained using this method is denoted as $\:{P}_{PEnhanced}\left(x,y\right)$.

## Mathematical model for super-resolution image generation

The mathematical details of the GF-CWAO-GAN Processing steps for shallow and deep feature extraction is described in the section below:

### Generator

The generator network comprises several layers named Input layer, Convolutional layer, activation layer and sub-pixel layer and their functionalities as detailed below.

### Input layer

The Patch Enhanced image $\:{P}_{PEnhanced}\left(x,y\right)$ is fed into the input layer of the generator where the image is read and regularized before being forwarded to convolution layer. The output produced by this layer, denoted as I_GAN_ is as below:

13 $$I_{GAN}=\;{regularise}({P}_{PEnhanced}\left(x,y\right))$$

Where, *regularise* brings comprehensibility to the patch enhanced image via *regularise()* function.

### Convolution layer

The output of the regularized input layer undergoes characteristic/feature mapping in this convolution layer and therefore the resultant output of this convolutional layer denoted as ConvL_*GAN*_ is shown obtained as indicated below:

14 $$\:{ConvL}_{GAN}=\zeta\:\left({\omega\:}^{o}*{I}_{GAN}+B\right)$$

Where, $\:{\omega\:}^{o}$ signifies the hidden layer weight value that is being optimized using AO function and $\:\zeta\:$and B denotes the kernel size and bias value.

### Activation layer

The usage of activation layer in the generator model helps detect the hidden patterns in the image. In this layer, the only characteristic features are extracted using Parametric ReLu (PReLu) activation function, $\chi$, expressed in Eq. (15) below is designed to further optimize generator neural network performance. PReLu is an advanced variation of traditional Leaky ReLu and ReLu that is designed for improving optimization performance of neural networks. The resultant output of activation layer denoted Activation_*GAN*_ is as given below:

15 $$Activation_{GAN} =\:\chi\:\left({ConvL}_{GAN}\right)=\left\{\begin{array}{c}{ConvL}_{GAN},\:\:\:\:\:\:\:\:\:if{ConvL}_{GAN}>0\\\:w{ConvL}_{GAN},\:\:\:\:if{ConvL}_{GAN}\le\:0\end{array}\right\}$$

Where, w is the ReLu constant.

Sub-pixel Convolution layer: In this layer, mutli-channel features are extracted from ActivationGAN followed by merging operation for generation of SR image. The sub-pixel convolution layer output, denoted as $\:\left({\widehat{SPL}}_{GAN}\right)$ is,

16 $$\:{\widehat{S}PL}_{GAN}=\varPsi\:\left({Activation}_{GAN}\right)$$

Where, $\:\varPsi\:$ is the merging function. Thus, the resultant SR image generated is represented as $\:{G}_{SR}\left(x,y\right)$.

### Discriminator

Remote sensing images comprise of rich texture and ample amount of information in it which can be used to discern the image originality of the rebuilt HR remote sensing images which in turn help in improving the quality of the image. This article proposes Gompertz Function (GF) layer in the discriminator model of GF-CWAO GAN model. The GF layer outputs the prominent value of each discrete image component(pixel) in the image and thus improve the capability of GF-CWAO GAN model to distinguish the image details.

Further, the generated image from the generator is passed through the discriminator where originality of the image is verified through the series of layers stacked like convolution layer, activation layer, matching layer and GF layer. The process begins with feature mapping at convolution layer followed by active feature extraction. The resulting feature points are fed into the matching layer at which the following task takes place.

### Initial matching

This stage is determined to establish homography relationship between the resulting features and is denoted by

17 $$\:{H}_{SR}=\frac{1}{p}\left({\sum\:}_{z}{P}_{SR}^{Yi}\left(x,y\right){P}_{SR}^{Yj}\left(x,y\right)\right)$$

Where, z denotes p number of active features and (Y_i_, Y_j_) refers to the initial matching points.

### Propagation matching

Since the initial matching, also called shallow feature mapping, does not cover the entire feature points in the generated image, propagation matching also referred to as deep feature mapping is done that involves two different steps probability relaxation and geometric correspondence matching respectively. The features points that were not matched in the initial matching stage are selected and the homography relationship is determined between the initial matching output and the selected feature points. In probability relaxation matching, the candidate matching features are computed for the exactly matched feature points. The probability of the exact matched feature points, denoted as $\:\left({\widehat{H}}_{abs}\right)$, is specified as,

18 $$\:{\widehat{H}}_{abs}=\varOmega\:\left({H}_{SR}\right)$$

Where, $\:\varOmega\:$ is the probability function and the matching layer output becomes $\:{\widehat{M}}_{SR}$.

GF Layer: Among the matching feature points, only the contributing feature points are selected in this layer by using Gompertz Function $(GF_{SR})$ by avoiding convergence problem and is denoted as below:

19 $$GF_{SR}=Fe^{-ge^-{h\tau}}$$

Where, *F* means an asymptote, *g* is the displacement vector and *h* mean the growth rate at time τ. Finally, the discriminator provides the output as real or fake 

and is denoted as$\:{C}_{DSR}\left(x,y\right).$ 

### Convergence War AO (CWAO)-based weight optimization

War strategy optimization is a meta-heuristic optimization algorithm that is based on strategic movement of army troops during the war. The war strategy is modelled such that each soldier dynamically reaches the optimal value as a part of optimization process. Weight updating mechanism and weak soldier relocation strategy is followed to improve convergence and robustness of the model^25^. War strategy optimization is also used for efficient scanning electron microscopy image segmentation through effective optimized weights^26^.

In the proposed architecture, the war strategy optimization idea is incorporated as weight updating mechanism during the training process to achieve the local optimum. Hence Convergence Accelerometric Function (CAF) is proposed for weight adjustment for achieving the global optimal solution by backpropagation method while training the model. This amalgamation of CAF with the traditional WSO is termed as Convergence War Accelerometric Optimization (CWAO). This process is detailed below.

Phase 1: Initially, the weight values $(\omega^{\widehat{G}})$ are randomly distributed on the nodes as follows:

20 $$\omega^{\widehat{G}}=\left\{{\omega^{1},\omega^{2},\omega^{3},...,\omega^{N}}\right\}$$

Then, the generation accuracy (fitness) of each is determined and the one with the better fitness value is selected as king. The fitness determination $(f(\omega^{\widehat{G}}))$ is represented as equation (21).

21 $$f(\omega^{\widehat{G}})=\max_{accuracy}(\omega^{\widehat{G}})$$

Phase 2: Further, the weight of every node is updated depending on the outcome of the current weights. If the image super-resolution improves, then based on the strategy success, the weights of all the nodes are varied. Thus, the updated weights of the nodes become,

22 $$\xi(\omega^{\widehat{G}}(t+1))=\xi(\omega^{\widehat{G}}(t))+2\times\varphi\times(\vartheta-\rho)+rnd$$

Here, $\xi(\omega^{\widehat{G}}(t+1))$ determines the updated weight, $\varphi$ signifies the earlier weight and $\vartheta-\rho$ defines the position of the nodes.

Phase 3: Based on the updated image, the weights are also updated. If the current weight (E_n_) is closer to the earlier weight (E_w_), then the update equation is as given below:

23 $$\xi(\omega^{\widehat{G}}(t+1))=\xi(\omega^{\widehat{G}}(t+1))+(E_n\geq\,E_{\stackrel{-}{w}}+\xi(\omega^{\widehat{G}})$$

Phase 4: Next as a defence strategy, the position of the nodes in the network is updated. During every phase, the diminishing nodes (that worsen the image quality) are identified and replaced using the CAF as given in the equation below:

24 $$\xi(\omega^{\widehat{G}}+1)=RND\times\frac{\text{(}{\stackrel{-}{w}}{best}-{\stackrel{-}{w}}_{ff})}{{f}({\stackrel{-}{w}}_{best})+\upsilon}$$

Where, RND refers to the randomly distributed random variable, ${\stackrel{-}{w}}_{best}$ is the best weight, and $\upsilon$ is the CAF. The above process is repeated until the optimal weights are obtained and is represented as $\omega^\circ$ . The proposed CWAO pseudocode is presented below.
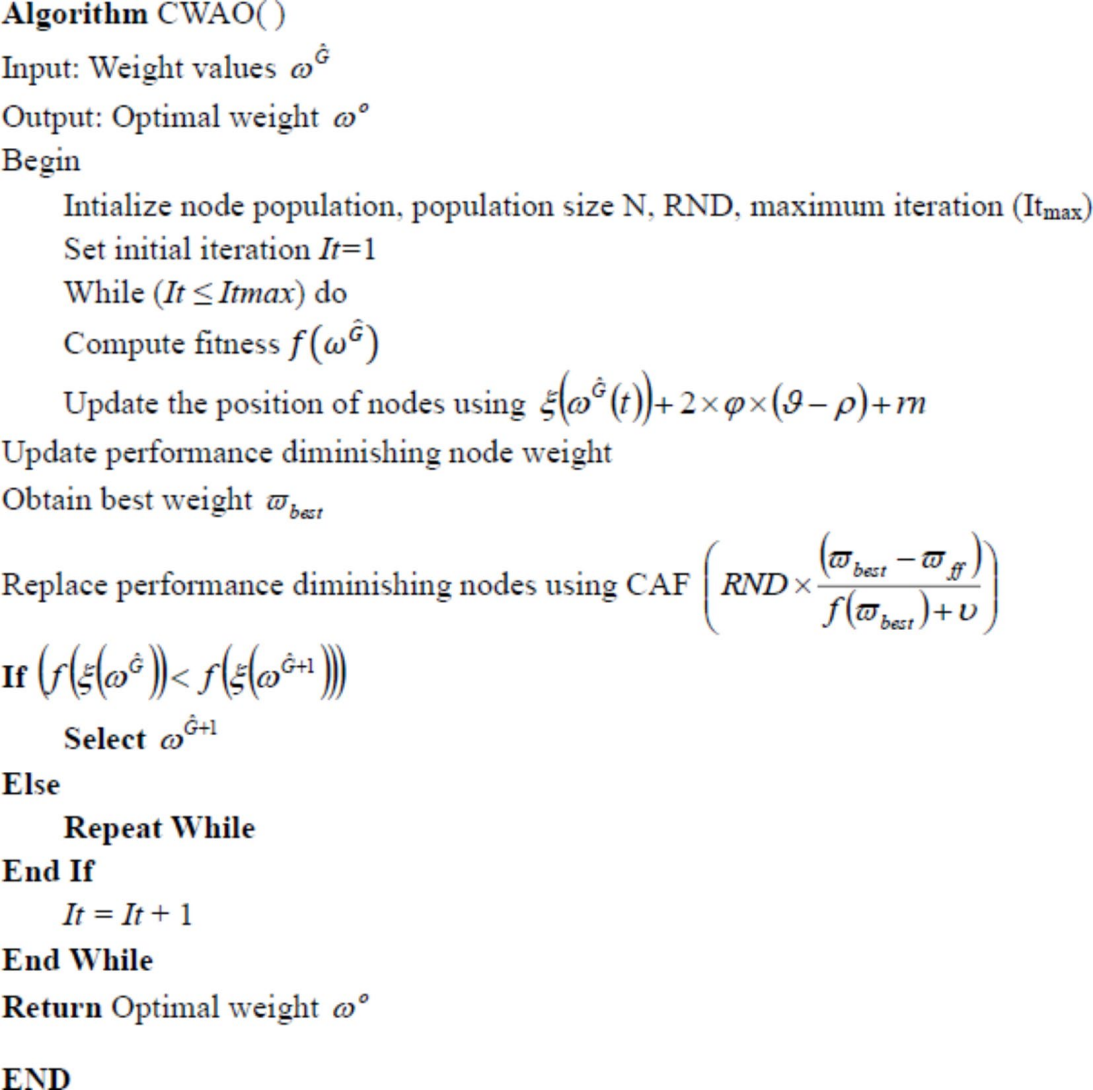


## Experiments

### Data preparation

The random 128 × 128 pixel crops are extracted from the HR image training dataset. Data Augmentation was applied by randomly flipping the cropped images horizontally, generating around 20 more training samples from each image and following it with normalization to the range [0, 1] which helped prevent over-fitting^27^.

### Training details

Model training is carried out in dual stages: a PSNR-oriented stage and GF-CWAO-GAN stage. Each stage focused on enhancing different aspects of image SR task. A final Interpolation Model was obtained by taking the weighted average values of both the models.

#### PSNR – oriented training

The primary focus of this stage prioritized to attain a high Peak-Signal-to-Noise-Ratio (PSNR) as an objective measure of image quality. The details pertaining to this are as follows:

- **Loss Function**: L1 pixel loss was considered as the primary loss function that calculates the pixel wise difference between the generated HR image and the ground truth image. Lower the loss value indicates superior performance thereby resulting in improved PSNR metrics^6^.

25 $$\:MAE=1/n{\sum\:}_{i=1}^{n}|\text{y}\text{i}-{\widehat{y}}i\:\:|$$

Here, yi is the ground truth image value; yˆi is the predicted value; n is the number of samples.

- **Optimizer and Learning Rate Schedule**: Here ADAM optimizer is applied with initial LR of 2 × 10^−4^. The incorporation of multi-step scheduler diminished the learning rate by a factor of 0.5 i.e., half at iterations [1000, 3000, 5000, 7000]^9^.
- **Training Parameters**: The model was trained for 8000 iterations with a batch size of 16.
- **Check-Pointing**: Model check points were saved every 1000 iterations that allowed the model to train with limited GPU capabilities by pausing and resuming in two cycles of 4000 iterations each.

#### GF-CWAO-GAN training

The image taken from the dataset undergoes patch extraction as discussed in Sect. 3.3 followed by patch clustering step. Patch Clustering is done by applying KL-KM-KMA. KMA is the most widely used clustering approach due to its better clustering efficiency in reduced time^28^^[,29^. Nonetheless, the Euclidean distance-based distance measurement affected the clustering accuracy. Hence, the Krzanowski and Li Index (KLI) and the Kantorovich Metric (KM) are applied for discrepancy/distance measurement, used to describe the similarity between the real and generated images. This combination of KLI and KM to the conventional KMA is termed as KLI-KM-KMA is discussed below.

The patches extracted from remote sensing SR $\:\left({R}^{c}\right)$ is given as input to KLI-KM-KMA and clustered into N number of cluster centers $\:\left({\stackrel{-}{C}}^{N}\right)$. Here the cluster centres are randomly selected, and the number of clusters are selected using KLI$\:\left({\stackrel{-}{C}}_{KLI}^{N}\right)$ as detailed below.

26 $$\:{\stackrel{-}{C}}_{KLI}^{N}=\left|\frac{diff\left({R}^{c}\right)}{diff\left({R}^{c-1}\right)}\right|$$

Where, $\:diff\left({R}^{c}\right)\:and\:diff\left({R}^{c-1}\right)$ defines the within group dispersion of the c^th^ and c-1th feature.

Then, the distance between the input $\:\left({R}^{c}\right)$ and the selected cluster centroid $\:\left({\stackrel{-}{C}}^{N}\right)$ is evaluated by KM function $\:\left(k\left({R}^{c},{\stackrel{-}{C}}^{N}\right)\right)$, defined as,

27 $$\:k\left({R}^{c},{\stackrel{-}{C}}^{N}\right)=SUP\left\{\int\:{R}^{c}dc-\int\:{\stackrel{-}{C}}^{N}.{R}^{c}dc\right\}$$

Where, SUP demonstrates the real-valued function.

Further, the extracted patches are clustered to the nearest centroid with a minimal central difference. Hence the objective function ($\:\mathcal{F}$) of KLI-KM-KMA can be defined as,

28 $$\:\mathcal{F}={{\sum\:}_{c=1}^{e}{\sum\:}_{N=1}^{l}\left|k\left({R}^{c},{\stackrel{-}{C}}^{N}\right)\right|}^{2}$$

The above steps are repeated until the cluster centres remain unchanged. Therefore, the final ${\stackrel{\sim}{m}}-$ number of patch clusters formed using KLI-KM-KMA $\:\left({\nu\:}^{p}\right)$ is represented as the following set as given below,

29 $$\:{\nu\:}^{p}=\left\{{\nu\:}^{1},{\nu\:}^{2},{\nu\:}^{3},{\nu\:}^{4},...,{\nu\:}^{\stackrel{\sim}{m}}\right\}$$

The pseudocode for the proposed KLI-KM-KMA is presented as below,
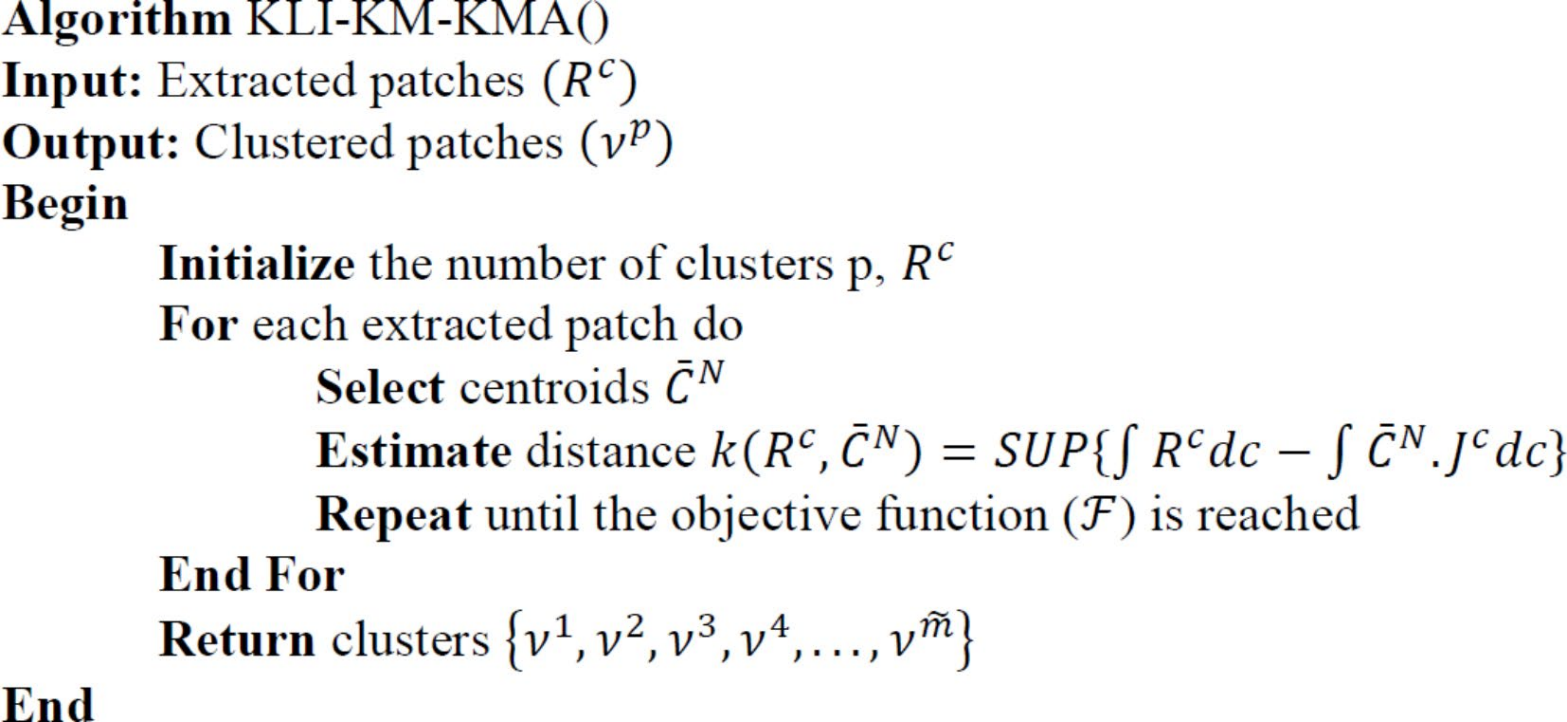

- **Generator Initialization**: The proposed work implemented Transfer Learning via initializing the generator here by loading the weights from the PSNR-Oriented model.
- **Discriminator Network**: The Discriminator Network was trained adversarial alongside the Generator Network. PatchGAN architecture network was used in discriminator that guide in distinguishing between real and generated HR images.
- **Loss Function**: The loss function used here during training is a combination of L1 Pixel Loss (Mean Absolute Error) and VGG (Perceptual Loss).
- **L1 Pixel Loss**: Assigned a lower weight i.e., 1 × 10^−2^ to maintain a minimum image quality.

30 $$\:\text{W}\text{e}\text{i}\text{g}\text{h}\text{t}\text{e}\text{d}\:\text{L}1\:\text{P}\text{i}\text{x}\text{e}\text{l}\:\text{L}\text{o}\text{s}\text{s}=1\:\text{x}\:{10-}^{2}\:x\:1/N{\sum\:}_{i=1}^{n}|\text{y}\text{i}-{\widehat{y}}i\:\:|$$

- **VGG Feature Loss (Perceptual Loss)**: Pretrained VGG model was used with the weight assignment of 1.0 for feature maps extraction. This loss compares the loss in the feature maps extracted for both generated HR and ground truth images.

31 $$VGG \;Feature \;Loss\;\:=\:\lambda\:\:\mathsf{{\rm\:X}}{\sum\:}_{i=1}^{N}\left|\right|{\upphi\:}\text{i}({\widehat{y}})-\:{\upphi\:}\text{i}\left(\text{y}\right)\left|\right|2$$

Where, $\:\lambda\:$ is the weight assigned as 1.0; $\:{\upphi\:}\text{i}({\widehat{y}})$ - the feature maps extracted from the generated image ${\widehat{y}}$ by the VGG model; $\:{\upphi\:}\text{i}\left(\text{y}\right)$ – the feature maps extracted from the ground truth image y; N – total number of layers in the VGG model.

- **Optimizers and LR Schedule**: Like the earlier training, ADAM optimizers were used for both generator and discriminator with the difference being different LR Schedules for each network. The initial LR was set to 1 × 10 − 4 for both, with a decay factor of 0.5 i.e., half with multistep scheduler at iterations [500, 1000, 2000,3000].
- **Training Parameters**: Trained the model for 4000 iterations with a batch size of 16. Here the number of iterations were reduced since this model is built upon the earlier trained weights from the PSNR-Oriented model.
- **Check-pointing**: While training this model, check points were saved every 500 iterations, and the training was carried out along 2 cycles comprising 2000 iterations each.

#### Network interpolation

Though both PSNR-Oriented and GF-CWAO-GAN model have provided remarkable results as individual models, yet it implemented network interpolation by taking weighted average of both the models to improve visual perception of image quality. The interpolation factor, α, varied to control the weight of the model where α was set to 0.8 (higher) for GF-CWAO-GAN model while taking influence from PSNR-Oriented model too. Both models used ADAM optimizers with β1 and β2 set to 0.9 and 0.99 values for PSNR-Oriented and GF-CWAO-GAN model respectively – implemented using Tensorflow and trained on Colab’s TPU offerings.

## Results and discussion

This segment discusses the corresponding results obtained from the experiments conducted as mentioned in earlier section using the dataset mentioned below under section A.

### Dataset

The WorldStrat dataset is used for training, SR image generation and evaluation. This dataset is commonly used in deep learning for remote sensing image SR. This dataset originally contains HR images of 1.5 m/pixel which were resized into dimensions [512,512] and around 405 such HR images are considered for experiment. The images were downsampled by applying Lanczos resampling during this process and resized into LR images of dimensions [128, 128] for testing the model. Further, the downsampled images are randomly assigned to the training set (80%) and the test set (20%).

#### Evaluation metrics used

The performance of the proposed model was evaluated using the following metrics. Peak Signal-to-Noise Ratio (PSNR), Structural Similarity Index Measure (SSIM), Inception Score, Fréchet Inception Distance, Accuracy, Precision and Sensitivity.

#### Quantitative evaluation

Quantitative evaluation was carried out using four metrics mentioned above in sub section B of Sect. 5. Table II below shows the PSNR and SSIM scores achieved for GF-CWAO-GAN, Medi-ESRGAN, SRGAN and ESRGAN. It can be observed that GF-CWAO-GAN outperforms over the other models on all images, both in terms of PSNR and SSIM metrics.

**Fig. 5:**
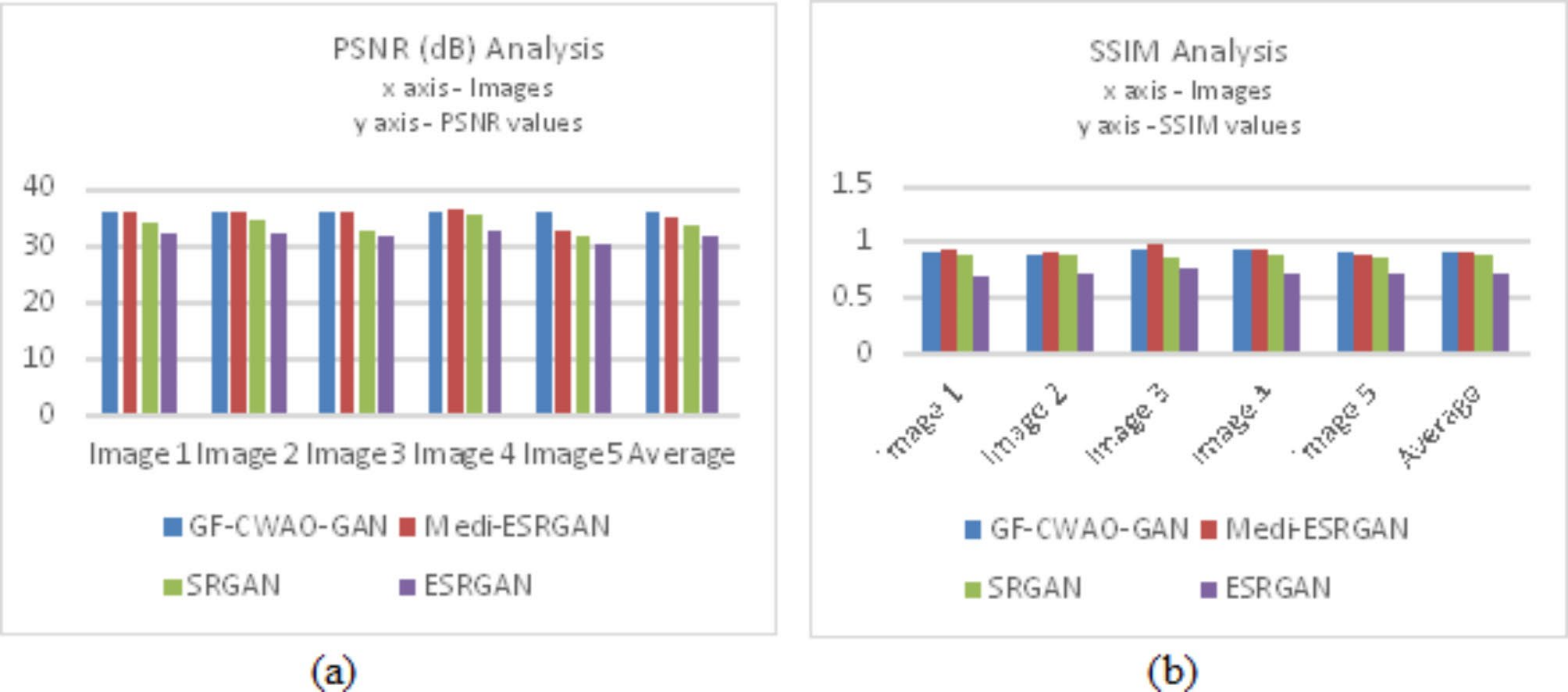
Performance analysis of proposed model for parameters (**a**) PSNR and (**b**) SSIM.

As shown in Table 1, it can be observed that the results of the four GAN-based SR models for PSNR and SSIM are calculated for 5 images and averaging them (as shown in last column and corresponding graph in Fig. 5 below) across multiple images to obtain reliable and robust measure of overall performance of proposed SR model by smoothing out individual variability, thereby facilitating consistent comparison with other state-of-the-art models. It is observed that GF-CWAO-GAN (35.86dB, 0.903) and Medi-ESRGAN (34.83dB, 0.896) have outperformed PSNR and SSIM parameter values respectively. The good performance of GF-CWAO-GAN model expresses that it is effective and feasible to use this model for SR reconstruction of remote sensing hyperspectral images.

**Table 1:** Quantitative evaluation of proposed model with other gan models based on psnr and ssim.

PSNR (dB)	Image 1	Image 2	Image 3	Image 4	Image 5	Average
GF-CWAO-GAN	35.97	35.62	35.93	35.91	35.88	35.86
Medi-ESRGAN	35.89	35.89	35.59	36.1	32.66	34.83
SRGAN	33.93	34.37	32.57	35.35	31.44	33.53
ESRGAN	31.85	31.97	31.42	32.23	30.08	31.51
**SSIM**	**Image 1**	**Image 2**	**Image 3**	**Image 4**	**Image 5**	**Average**
GF-CWAO-GAN	0.892	0.878	0.932	0.916	0.897	0.903
Medi-ESRGAN	0.912	0.908	0.971	0.918	0.872	0.896
SRGAN	0.871	0.868	0.844	0.882	0.854	0.864
ESRGAN	0.671	0.717	0.748	0.705	0.714	0.711

We also correlated the proposed model with the other three state-of-the-art models’ perceptual image quality, training time and resource requirements as shown in Table 2 below. The training time was measured from the start of the training stage until the point of convergence where the predefined validation loss threshold is reached. Our model demonstrated a significant reduction in training time, completing the training stage in 6–7 h compared to the other reference/guiding models like ESRGAN and SRGAN. This improvement in training time is attributed to the use of CWAO weight optimization, ADAM optimizer and LR schedule in turn providing enhanced efficiency. All the experiments are implemented on Colab T4 GPU (16GB VRAM) to combat the computationally expensive physical resource limitations.

**Table 2:** Comparison of different gan models.

Model	Dataset	Image Quality (PSNR/SSIM)	Training Time	Resource Requirements
GF-CWAO-GAN	WorldStrat	35.86/0.903	6–7 h	Colab T4 GPU (16GB VRAM)
Medi-ESRGAN	STARE (8000 + 4000)	34.83/0.896	6–7 h	Colab T4 GPU (16GB VRAM)
SRGAN	CelebA/10	33.53/0.864	~ 10 h	NVIDIA GTX 1080 Ti (11GB VRAM)
ESRGAN	Div2k/100	31.51/0.711	12–24 h	NVIDIA RTX 3080 Ti (10GB VRAM)

The performance of the proposed approach is computed for evaluation metrics Fréchet Inception Distance (FID), Inception Score (IS), Accuracy, Precision and Sensitivity. Table 3below unveils the comparative measure of the proposed technique in terms of IS and FID. As per the studies, while the original paper ESRGAN has not reported IS and FID values, the other literature have indicated that higher the values of IS and lower the FID, the better the GANs are suited for image generation^10,11^. From Table 3, it can be noticed that the proposed model surpasses with the IS value of 8.71 (higher) and FID value of 36.4 (lower) over the GANs and CNNs/DNNs. Thus, the proposed method generates noise free SR images when compared to other traditional methodologies.

**Table 3:** Comparitive measure of IS and FID.

Model	Inception Score (IS)	Fréchet Inception Distance (FID)
GF – CWAO - GAN	8.71	36.4
Improved GAN	8.09	37.7
DNN/CNN	6.16	29.3

**Fig. 6:**
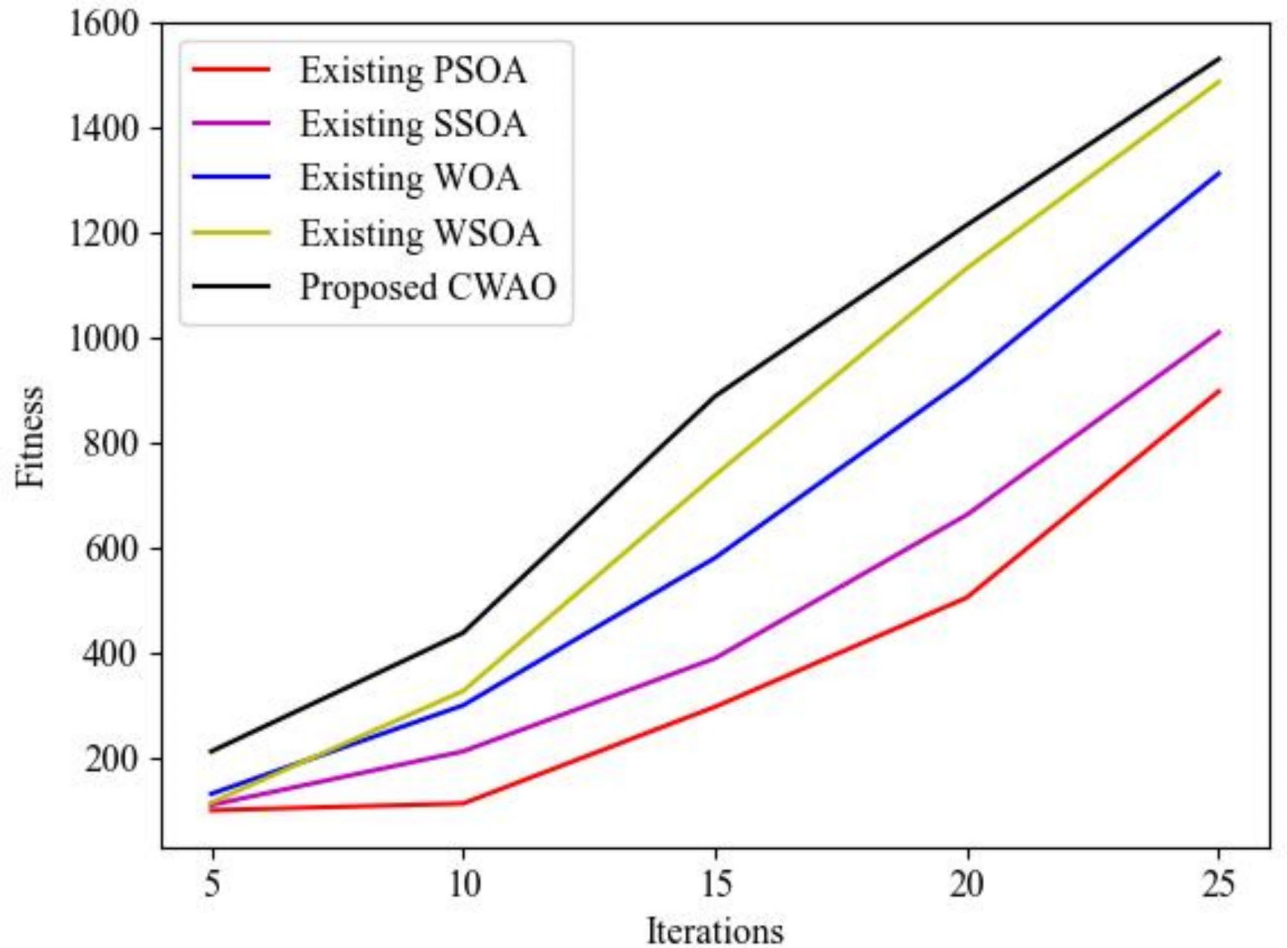
Fitness performance measure for the proposed technique.

Following further, the performance measure of the proposed CWAO in terms of Fitness (as shown in Fig. 6 below) is presented to substantiate its superiority while comparing it with the existing Particle Swarm Optimization Algorithm (PSOA), Salp Swarm Optimization Algorithm (SSOA), Whale Optimization Algorithm (WOA) and White Shark Optimization Algorithm (WSOA). Fitness is typically evaluated using the combination of IS, FID and perceptual quality metrics (i.e., SSIM) to provide comprehensive assessment of quality of generated images guiding optimization of proposed model during training process.

The CWAO incorporation into the proposed model achieves better performance gains by preventing premature convergence that is prevalent in basic WOA and PSOA. However, the usage of Gompertz function-based GAN permits CWAO to remain stable while learning high-frequency details crucial to SR tasks. CWAO converges faster for SR task when compared to SSOA, WSOA and WOA when handling large datasets validating the efficiency of Image SR task.

Advancing further, performance analysis of the proposed KL-KM-KMA in terms of clustering time with the other state-of-the-art clustering algorithms like K-Means (KMA), Fuzzy C Means (FCM), Mean Shift (MS) and Balanced Iterative Reducing and Clustering using Hierarchies (BIRCH) is made and is tabulated in Table 4 as below.

**Table 4:** Performance analysis of kl-km-kma with other state-of_the_art clustering algorithms.

Model	Clustering Time (ms)
Proposed KL-KM-KMA	16,523
KMA	24,879
FCM	32,543
MS	5980
BIRCH	7214

It is clear from the above Table 4 that the time taken to form clusters by the proposed approach is reduced (16523ms) compared to the conventional K-Means algorithm and FCM which confirms that the usage of KL-KM approach has avoided the issue of convergence hence improving the SR image generation performance. MS and BIRCH have lower Clustering timing against the compromise on other metrics.

**Fig. 7:**
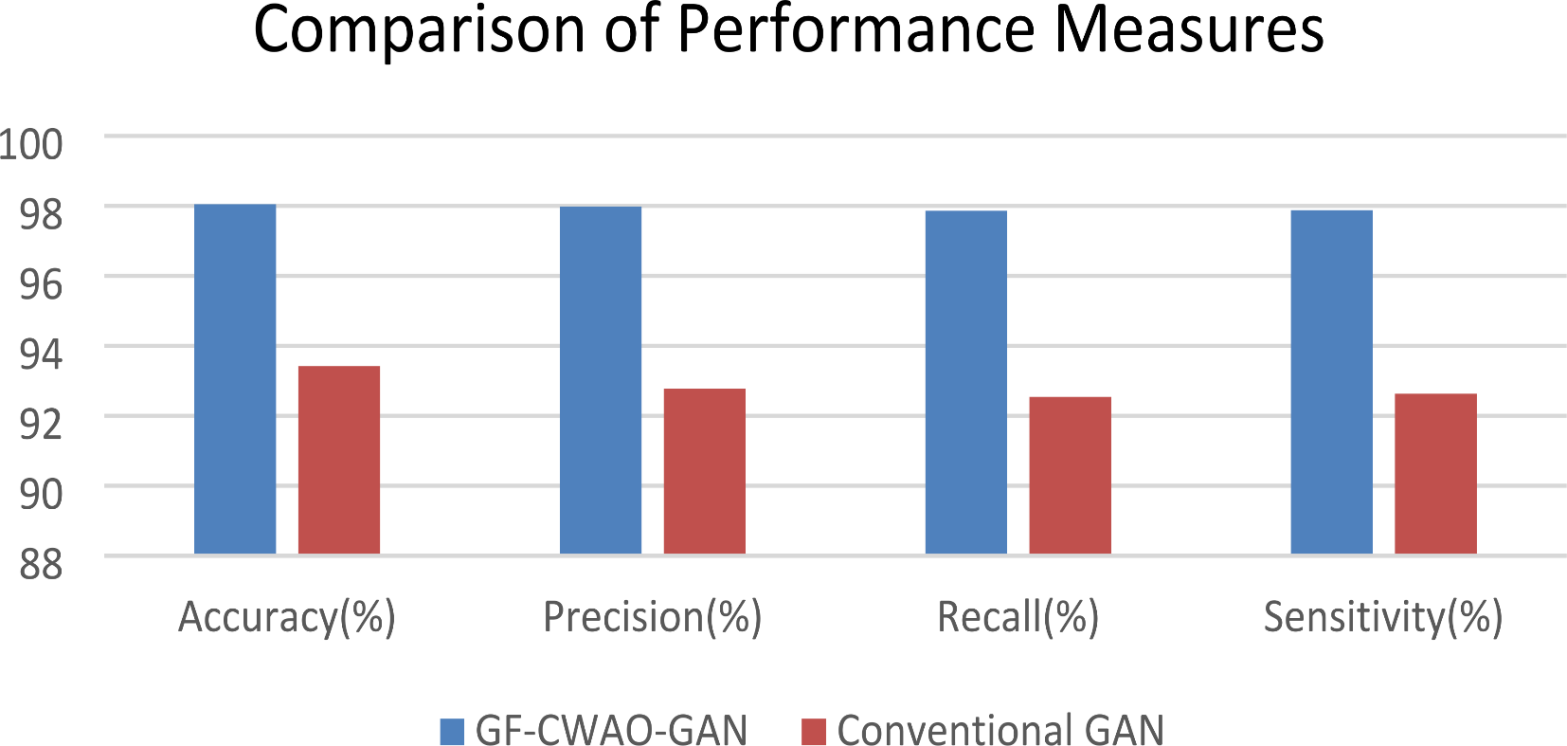
Comparison of Performance Measures for GF-CWAO-GAN and Conventional GAN.

The accomplishment of the proposed GF-CWAO-GAN approach with conventional GAN in terms of accuracy, precision, recall and sensitivity is shown in Table 5 and Fig. 7 below. It can be noticed that the proposed approach is superior in performance for all the metric listed above by the virtue of usage of GF and CWAO.

**Table 5:** Comparison of performance measures.

Model	Accuracy (%)	Precision (%)	Recall (%)	Sensitivity (%)
GF-CWAO-GAN	98.05	97.98	97.86	97.88
Conventional GAN	93.42	92.78	92.54	92.63

**Fig. 8:**
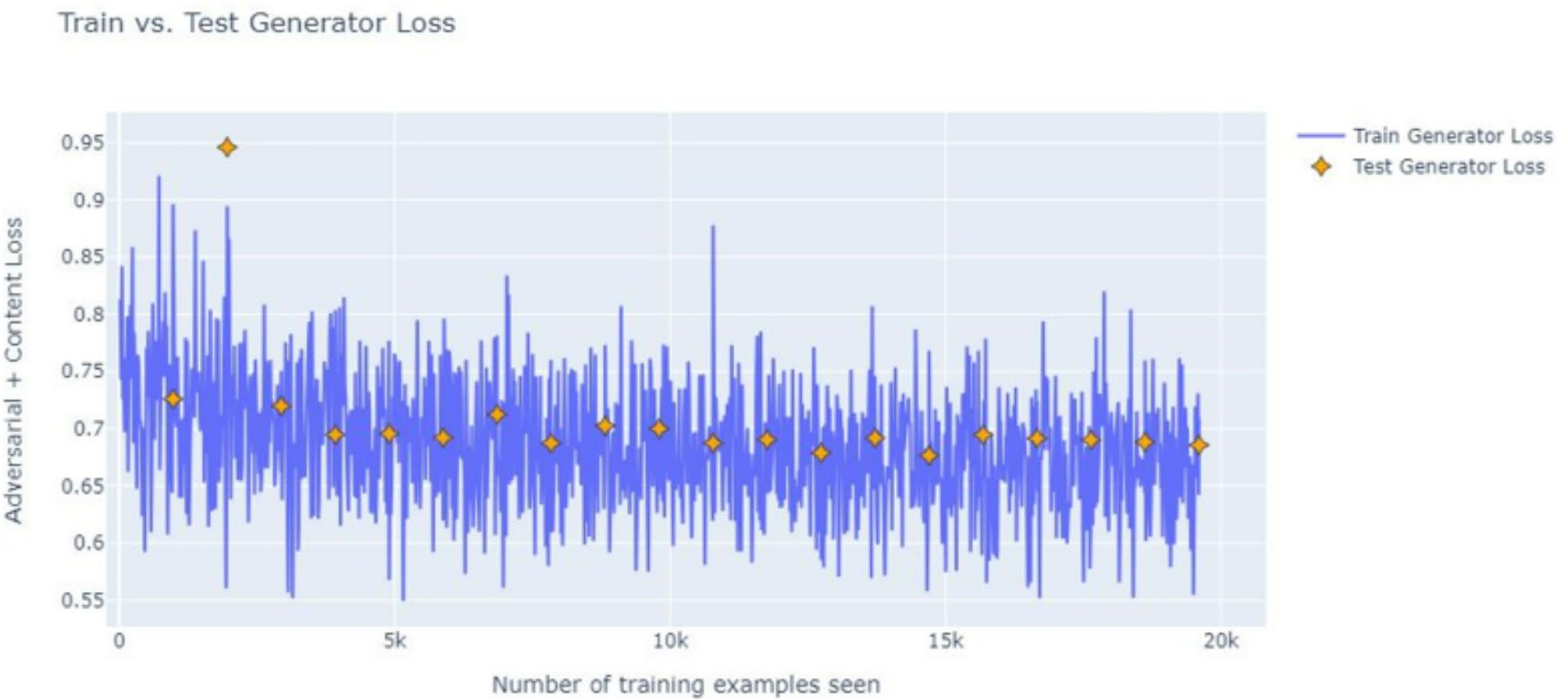
Training versus Test generator loss.

**Fig. 9:**
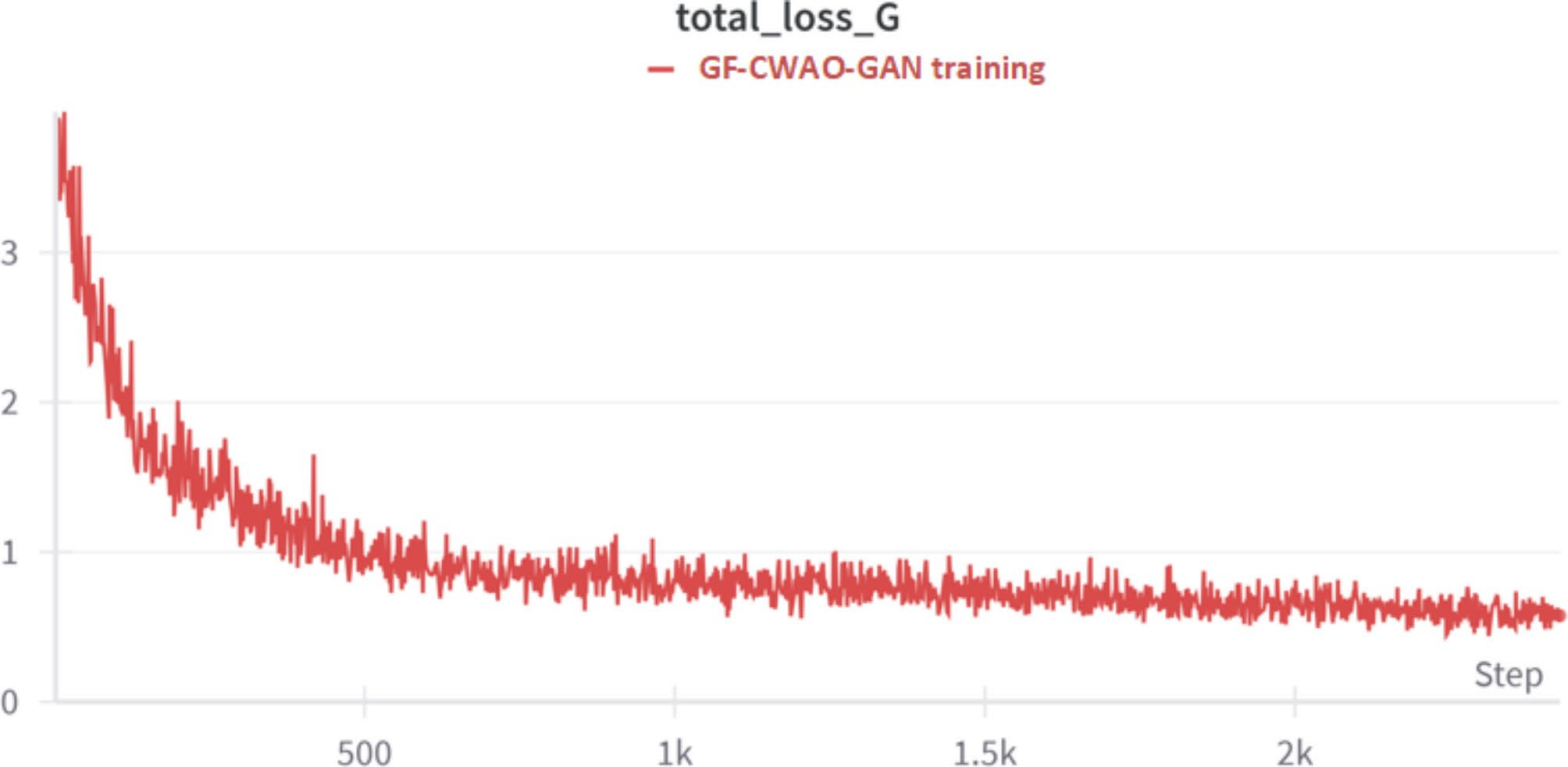
Total Loss.

Figure 8 below depicts the training versus test generator loss indicating that the training and test losses converge with the test loss being stable, indicating better generalization of model. Figure 9 below depicts the total generator loss curve decreasing over the training steps, indicating that GF-CWAO-GAN is successfully learning to generate realistic HR images. The fluctuations noticed in the plot are a result of adversarial training process. The stabilizing loss around step 1500 indicates that the model has entered a stable learning phase.

#### Qualitative evaluation

**Fig. 10:**
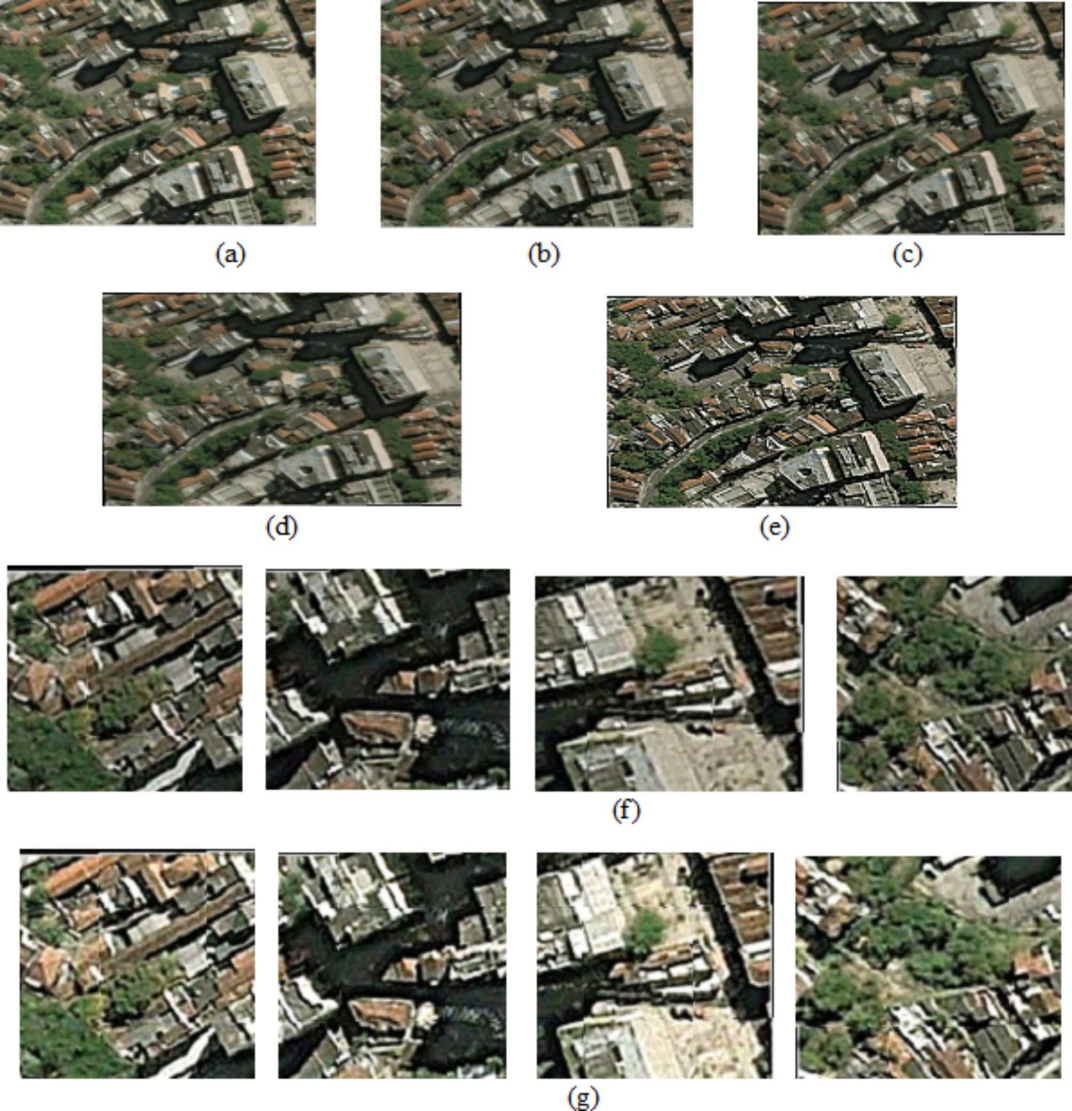
Sample image results for (**a**) Input image (**b**) Noise removed image (**c**)Tone mapped image (**d**) Skew corrected image (**e**) Boundary and edge enhanced image (**f**) Patch extraction (**g**) Patch enhancement.

**Fig. 11:**
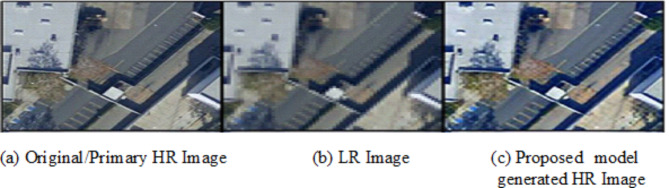
Sample Output 2.

**Fig. 12:**
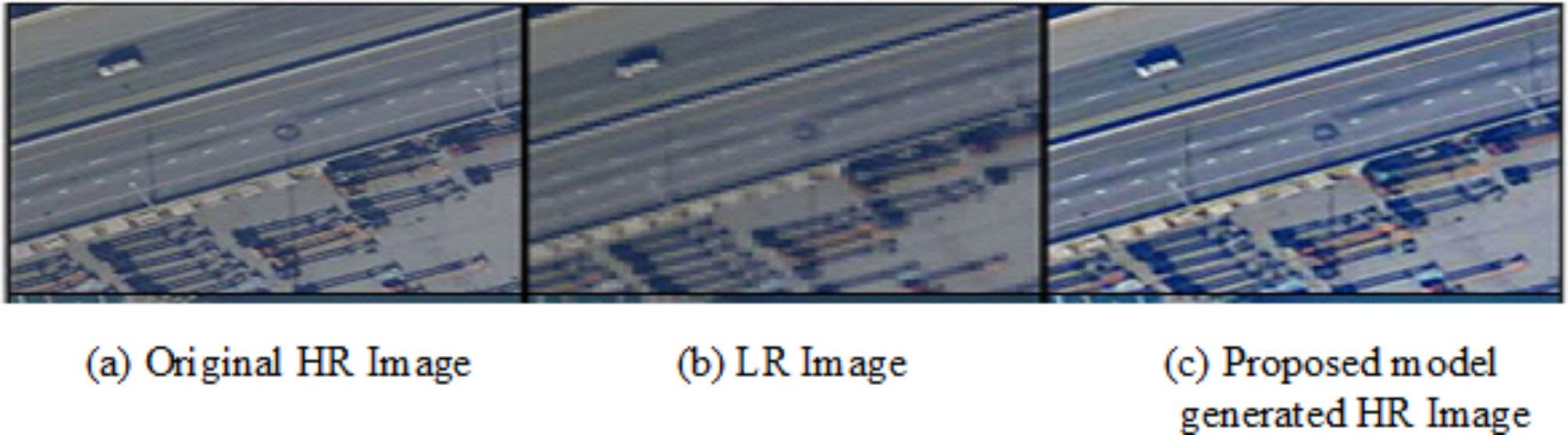
Sample Output 3.

Qualitative evaluation focusses on perceptual quality of SR image generated by the proposed model. It complements quantitative metrics like PSNR and SSIM to assess whether the generated images are realistic, sharp and visually appealing. The method focusses on restoring edges, textures and high-frequency information accurately, while avoiding blurriness. The higher Inception Score and lower Fréchet Inception Distance values achieved, and side-by-side visual comparisons are made as shown in figures below ensuring the visible improvements in the perceptual quality with respect to features like sharpness and texture consistency for LR Images, generated images and ground truth images. Visual images generated showed sharp edges with minimal artifacts that are closer to the ground truth image as shown in the Figs. 10, 11 and 12 below.

## Conclusion

In this paper, a novel deep learning-based unsupervised GF-CWAO-GAN is proposed for SISR of hyperspectral remote sensing image accomplished by training the model using KL-KM-KMA based clustering. Experiments were conducted on the benchmark dataset to show the performance and comparative analysis of the proposed model. It outperformed comparatively better over existing state-of-the-art GAN-based models both qualitatively and quantitatively with metric values, accuracy – 98.05%, precision – 97.98%, recall – 97.86%, PSNR – 35.86, SSIM – 0.903, Inception Score – 8.71, Fréchet Inception Distance – 36.4, clustering time – 16523ms and reduced model training time of 6–7 h. This work also proposed a solution to mitigate the necessity of expensive hardware and computational resources thereby improving SR image quality by utilizing more readily available resources. Even though the integration of Gompertz function to the model for Image SR has introduced certain merits like better convergence, quantitative metrics, reduced training time and learning rate, nonetheless it has introduced limitations like slow initial training, generalization issues with diverse datasets, reconstruction accuracy of complex textures, optimization and resource requirements that could be overcome by exploring adaptive mechanisms to mitigate these shortcomings.

## Data Availability

The data that support the findings of this study are available with the author deepthi.k@nmit.ac.in. and on reasonable request, the datasets are provided to the readers.

## References

[1] Bai, F., Lu, W., Huang, Y., Zha, L. & Yang, J. Densely convolutional attention network for image super-resolution. *Neurocomputing* **368**, 25–33 (2019).

[2] Shi, W. et al. Real-time single image and video super-resolution using an efficient sub-pixel convolutional neural network. arXiv:1609.05158 [cs.CV].

[3] Guo, D., Xia, Y., Xu, L., Li, W. & Luo, X. Remote sensing image super-resolution using cascade generative adversarial nets, Neurocomputing, Volume 443, Pages 117–130, ISSN 0925–2312, (2021). 10.1016/j.neucom.2021.02.026

[4] Ledig, C. et al. Photo-realistic single image super-resolution using a generative adversarial network. *IEEE Conf. Comput. Vis. Pattern Recognit.* 105–114. (2017).

[5] Wang, P., Zhang, H., Zhou, F. & Jiang, Z. Unsupervised Remote Sensing Image Super-Resolution Using Cycle CNN, *IGARSS 2019–2019 IEEE International Geoscience and Remote Sensing Symposium*, Yokohama, Japan, pp. 3117–3120, doi: (2019). 10.1109/IGARSS.2019.8898648

[6] Dong, X. et al. Remote Sensing Image Super-Resolution Using Novel Dense-Sampling Networks, in IEEE Transactions on Geoscience and Remote Sensing, vol. 59, no. 2, pp. 1618–1633, Feb. doi: (2021). 10.1109/TGRS.2020.2994253

[7] Xu, L., Zeng, X., Huang, Z., Li, W. & Zhang, H. Low-dose chest x-ray image super resolution using generative adversarial nets with spectral normalization. *Biomed. Sig Process. Control*. **55** https://doi.org/10.1016/j.bspc.2019.101600 (2020).

[8] Jia, S., Wang, Z., Li, Q., Jia, X. & Xu, M. Multiattention Generative Adversarial Network for Remote Sensing Image Super-Resolution, in IEEE Transactions on Geoscience and Remote Sensing, vol. 60, pp. 1–15, Art no. 5624715, doi: (2022). 10.1109/TGRS.2022.3180068

[9] Xiong, Y. et al. Improved SRGAN for remote sensing image Super-resolution Across locations and sensors. *Remote Sens.* **12**, 1263. 10.3390/rs12081263 (2020).

[10] Li, Y. et al. Single-Image Super-Resolution for Remote Sensing Images Using a Deep Generative Adversarial Network with Local and Global Attention Mechanisms, in *IEEE Transactions on Geoscience and Remote Sensing*, vol. 60, pp. 1–24, Art no. 3000224, doi: (2022). 10.1109/TGRS.2021.3093043

[11] Huang, Q., Li, W., Hu, T. & Tao, R. Hyperspectral Image Super-resolution Using Generative Adversarial Network and Residual Learning, ICASSP 2019–2019 IEEE International Conference on Acoustics, Speech and Signal Processing (ICASSP), Brighton, UK, pp. 3012–3016, doi: (2019). 10.1109/ICASSP.2019.8683893

[12] Yang, C. Y., Ma, C. & Yang, M. H. Single-image super-resolution: a benchmark. *Eur. Conf. Comput. Vis.* 372–386. (2018).

[13] Nasrollahi, K. & Moeslund, T. B. Super-resolution: a comprehensive survey. *Mach. Vis. Appl.* **25** (6), 1423–1468 (2017).

[14] arXiv:1406. 2661 [stat.ML]. 10.48550/arXiv.1406.2661

[15] Wang, C., Zhang, Y., Zhang, Y., Tian, R. & Ding, M. Mars Image Super-Resolution Based on Generative Adversarial Network, in *IEEE Access*, vol. 9, pp. 108889–108898, doi: (2021). 10.1109/ACCESS.2021.3101858

[16] Zhang, S. et al. Degradation learning for unsupervised hyperspectral image super-resolution based on generative adversarial network. *SIViP* **15**, 1695–1703. 10.1007/s11760-021-01902-9 (2021).

[17] Radford, A., Metz, L. & Chintala, S. Unsupervised representation learning with deep convolutional generative adversarial networks, in International conference on Learning Representations, ICLR, arXiv:1511.06434 [cs.LG] (2016).

[18] Wang, X. et al. Esrgan: Enhanced super-resolution generative adversarial networks. Proceedings of the European conference on computer vision (ECCV) workshops. (2018).

[19] Bezdek, J. C., Ehrlich, R. & Full, W. FCM: The fuzzy c-means clustering algorithm, Computers & Geosciences, Volume 10, Issues 2–3, Pages 191–203, ISSN 0098-3004, 10.1016/0098-3004(84)90020-7. (1984). https://www.sciencedirect.com/science/article/pii/0098300484900207).

[20] Ramin Ranjbarzadeh, S. B. & Saadi Automated liver and tumor segmentation based on concave and convex points using fuzzy c-means and mean shift clustering, Measurement, Volume 150, 107086, ISSN 0263–2241, 10.1016/j.measurement.2019.107086. (2020). https://www.sciencedirect.com/science/article/pii/S0263224119309522).

[21] Tian Zhang, R., Ramakrishnan, M. & Livny BIRCH: an efficient data clustering method for very large databases, ACM SIGMOD Record, 25, issue 2 Pages 103–114, 10.1145/235968.233324

[22] Agarap, A. F. Deep learning using rectified linear units (relu). arXiv preprint arXiv:1803.08375 (2018).

[23] Zhang, Y. et al. Image super-resolution using very deep residual channel attention networks. Proceedings of the European conference on computer vision (ECCV). (2018).

[24] He, K. et al. Deep residual learning for image recognition. Proceedings of the IEEE conference on computer vision and pattern recognition. (2016).

[25] Ayyarao, T. S. L. V. et al. War Strategy Optimization Algorithm: A New Effective Metaheuristic Algorithm for Global Optimization, in *IEEE Access*, vol. 10, pp. 25073–25105, doi: (2022). 10.1109/ACCESS.2022.3153493

[26] Amdjed Abdennouri, E. et al. An improved symmetric chaotic war strategy optimization algorithm for efficient scanning electron microscopy image segmentation: calcium oxide catalyst case, Chemometrics and Intelligent Laboratory systems, **244**, 105043, ISSN 0169–7439, (2024). 10.1016/j.chemolab.2023.105043

[27] Khosla, C. & Saini, B. S. Enhancing Performance of Deep Learning Models with different Data Augmentation Techniques: A Survey, 2020 International Conference on Intelligent Engineering and Management (ICIEM), London, UK, pp. 79–85. (2020).

[28] Sinaga, K. P. & Yang, M. S. Unsupervised K-Means Clustering Algorithm, in *IEEE Access*, vol. 8, pp. 80716–80727, doi: (2020). 10.1109/ACCESS.2020.2988796

[29] Gonzalez, R. & Woods, R. E. *Digital Image Processing, Up* (Saddle River Nj Pearson Hall, 2002).

